# Distribution of the genus *Boeckella* (Crustacea, Copepoda, Calanoida, Centropagidae) at high latitudes in South America and the main Antarctic biogeographic regions

**DOI:** 10.3897/zookeys.854.29614

**Published:** 2019-06-10

**Authors:** Claudia S. Maturana, Sebastián Rosenfeld, Javier Naretto, Peter Convey, Elie Poulin

**Affiliations:** 1 Laboratorio de Ecología Molecular, Departamento de Ciencias Ecológicas, Facultad de Ciencias, Universidad de Chile. Las Palmeras 3425, Ñuñoa, Santiago, Chile Instituto de Ecología y Biodiversidad Santiago Chile; 2 Instituto de Ecología y Biodiversidad (IEB), Las Palmeras 3425, Ñuñoa, Santiago, Chile Universidad de Chile Santiago Chile; 3 Laboratorio de Ecosistemas Marinos Antárticos y Subantárticos, Universidad de Magallanes, casilla 113-D, Punta Arenas, Chile Universidad de Magallanes Punta Arenas Chile; 4 ONG Costa Humboldt, Canónigo Madariaga 570, Ñuñoa, Santiago, Chile ONG Costa Humboldt Santiago Chile; 5 British Antarctic Survey (BAS), Natural Environment Research Council, High Cross, Madingley Road, Cambridge CB3 0ET, UK British Antarctic Survey, Natural Environment Research Council Cambridge United Kingdom

**Keywords:** Antarctica, Falkland/Malvinas Islands, freshwater ecosystems, sub-Antarctic islands, Patagonia

## Abstract

Copepods are present in numerous aquatic environments, playing key roles in food webs, and are thought to be useful indicators of environmental change. *Boeckella* is a calanoid copepod genus distributed mainly in the Southern Hemisphere, with 14 species reported at higher southern latitudes in South America and Antarctica. We present an updated database of these 14 species of *Boeckella* generated from a combination of three sources: 1) new field sampling data, 2) published records, and 3) Global Biodiversity Information Facility (GBIF), to provide a comprehensive description of the geographic distribution of the genus south of latitude 40°S in southern South America and the three main terrestrial biogeographic regions of Antarctica. The database includes 380 records, 62 from field sampling, 278 from the literature and 40 from GBIF. Southern South America, including the Falkland/Malvinas Islands, had the highest species richness and number of records (14 and 297, respectively), followed by the sub-Antarctic islands (5 and 34), South Orkney Islands (2 and 14), South Shetland Islands (1 and 23), Antarctic Peninsula (1 and 10) and finally continental Antarctica (1 and 2). *Boeckellapoppei* Mrázek, 1901 is the only representative of the genus, and more widely the only terrestrial/freshwater invertebrate, currently reported from all three main biogeographic regions in Antarctica (sub-Antarctic islands, maritime and continental Antarctic). Future development of molecular systematic studies in this group should contribute to assessing the correspondence between morphological taxonomy and molecular evolutionary radiation.

## Introduction

Knowledge of the diversity and distribution of organisms over space and time can provide information about changes in the composition of communities in different environments, particularly in sensitive ecosystems such as those in freshwater. Such information can also be used in biogeographic and niche modelling studies, contributing to understand the ecology of a given taxon. However, despite international efforts to increase the digitization of catalogues of specimens in museums and other repositories, even today only a small proportion of the total worldwide records are estimated to have been made available online through the efforts of the Global Biodiversity Information Facility (Ariño 2010).

Copepods are thought to be one of the most abundant metazoan groups in the world (Huys and Boxshall 1991), colonizing virtually all aquatic habitats (Bayly and Boxshall 2009) from the deepest ocean abyss (Bradford-Grieve 2004) to high mountain lakes in the Himalayas (Sommaruga 2010) and Andes (Zagarese et al. 1997), and from hydrothermal springs (Ivanenko 2006; Ivanenko and Defaye 2006) to the frozen lakes of Antarctica (Bayly et al. 2003; Convey et al. 2008). They play fundamental ecological roles, being key components of food webs in both marine and freshwater ecosystems, and in some cases being recognized as useful indicators of environmental change (Gerten and Adrian 2002; Hays et al. 2005). However, the lack of updated and accessible data limits the ability to assess the impact of environmental change on their diversity and distribution. Species of the order Calanoida have undergone considerable adaptive radiation and diversification. They inhabit a great variety of aquatic environments (Adamowicz et al. 2010), with tolerance of a wide conductivity gradient (De los Ríos et al. 2010). Although the number of freshwater species is considerable (21% of the total species described), the majority of diversity is present in the marine environment (Jaume et al. 2004). Because of this, most studies to date have focused on marine copepods.

*Boeckella* is a freshwater calanoid copepod genus that currently includes 42 described species restricted to the Southern Hemisphere (Bayly 1992a), with some discrete records of *B.triarticulata* (G.M. Thomson, 1883) from Mongolia and several introduced populations in Italy (Bayly 1992b; Ferrari and Rossetti 2006, Alfonso and Belmonte 2008). *Boeckella* is one of the most representative groups of calanoids in the freshwater ecosystems of southern South America, Australasia (Australia, New Zealand, Tasmania, New Caledonia) and various sub-Antarctic and cool temperate islands (Marion and Prince Edward Islands, Crozet Islands, Kerguelen Islands, Heard Island, Macquarie Island, Campbell Island, Amsterdam Island and South Georgia). *Boeckellapoppei* is the only calanoid species recorded in continental and maritime Antarctica (Bayly 1992b; Pugh et al. 2002; Bissett et al. 2005; Maturana et al. 2018).

Fourteen species of *Boeckella* have been reported from higher southern latitudes (beyond 40°S) in South America, including Patagonia and Tierra del Fuego, Falkland/Malvinas Islands, various sub-Antarctic islands and Antarctica (Pugh et al. 2002; Bayly et al. 2003). According to the latest taxonomic and phylogenetic studies (Bayly 1992b; Adamowicz et al. 2007), these 14 are considered taxonomically valid species (Walter and Boxshall 2018).

The present study provides an updated database of these 14 species of *Boeckella*, using a combination of recent sampling data, published records available in the literature and records from GBIF, giving a comprehensive description of the geographic distribution of the genus *Boeckella* at high latitudes in southern South America and the three main terrestrial biogeographic regions of Antarctica (sub-Antarctic islands, maritime and continental Antarctica; Convey 2013). This database will underpin future comprehensive systematic research on the genus, including the application of molecular phylogenetic approaches, allowing reconstruction of the regional evolutionary history of the genus, and in particular its members in the sub-Antarctic and Antarctic regions.

## Methods

### Data collation and construction of the database

The dataset (Maturana et al. 2018, https://doi.org/10.15468/zc6y59) was filtered by the area of interest, defined as South America at latitudes beyond 40°S, which encompasses most of the Patagonian and sub-Antarctic Provinces (Cabrera and Willink 1980; Morrone 2004; Sanches Osés and Pérez-Hernández 2005; Morrone 2006) and includes sub-polar forest and grassland ecoregions (Olson et al. 2001), along with the classically defined terrestrial biogeographic regions of Antarctica (Holdgate 1977; Convey 2017). The latter include the core sub-Antarctic islands (South Georgia, Prince Edward Islands, Macquarie Island, Heard Island, Crozet and Kerguelen Islands), maritime Antarctica (west coast of the Antarctic Peninsula, South Shetland Islands, South Orkney Islands, South Sandwich Islands) and continental Antarctica.

*Boeckella* records across this region were collated from three main sources: 1) recent field sampling data, 2) published literature and 3) data present in GBIF. Duplicate records were removed in combining these data to construct a unified database. To evaluate the quality of the collated data, all records were checked for mismatches between reported geographic location and the associated metadata, and taxonomically dubious records were excluded from the geospatial analysis.

Two main ecoregions in South America were considered for the purpose of geospatial analyses, the subpolar forest and grassland ecoregions as defined in the Terrestrial Ecoregions of the World (Olson et al. 2001) shape file (https://databasin.org/datasets/68635d7c77f-1475f9b6c1d1dbe0a4c4c; accessed 07/07/2018). Subpolar forest here includes the union of the Valdivian temperate forest and the Magallanes temperate forest ecoregions, and the grassland ecoregion includes the union of the Patagonian steppe and the Falkland/Malvinas Islands, which are on the continental shelf. The definitions of the continental, maritime and sub-Antarctic regions are as described in Convey (2017). All spatial analysis were carried out on the unified database.

### Recent sampling data

New material was collected from multiple locations in southern South America between Sierra Baguales in Chilean Patagonia (50°45.015'S; 72°25.158'W) and the Diego Ramirez archipelago (56°31.345'S; 68°43.622'W). In the Falkland/Malvinas Islands we collected from multiple ponds between Port San Carlos (51°27.690'S; 58°46.763'W) and North Arm (52°00.121'S; 59°17.407'W).

New Antarctic material was collected from the South Shetland Islands and Palmer Land in the southern Antarctic Peninsula under the framework of Antarctic Expeditions ECA53 and ECA54 of the Chilean Antarctic Institute (INACH). Samples from Alexander Island in the southern Antarctic Peninsula, South Orkney Islands and South Georgia were obtained during British Antarctic Survey (BAS) expeditions (2016–2017 and 2017–2018). Samples from Kerguelen and Crozet Islands were obtained under the PROTEKER project during the French Polar Institute Paul Emile Victor (IPEV) expedition (2017).

### Sample collection

Collections were made from the shoreline, scooping individuals from the water column of lakes, ponds and small pools using a zooplankton net (200 μm pore diameter) at locations across sub-Antarctic islands (Crozet, Kerguelen and South Georgia), maritime Antarctic (i.e. west side of Antarctic Peninsula, South Shetland Island and South Orkney Islands), part of the sub-polar forest ecoregion and Falkland/Malvinas Islands. Samples were immediately preserved in ethanol (99%), except for a small number of collected specimens that were preserved using formalin (5%) for morphological analysis. GPS positions were recorded for each sample location.

### Taxonomic identification

Morphological observations were performed under a stereomicroscope (LEICA EZ4) at 3.5× magnification. For determination to species level, the fifth leg was removed from male specimens and observed under an inverted microscope at 10× and 20× for confirmation of diagnostic characters as described by Bayly (1992a, 1992b) .

### Published literature

All available information was collated from the scientific literature reporting sampling or taxonomic revision of *Boeckella* species in southern South America, the sub-Antarctic islands, maritime and continental Antarctica. We included *Boeckella* records from 1855 to 1997 listed in the historical review of Menu-Marque et al. (2000), and additional information available in the literature from 1997 to present. Only records including the geographic location (coordinates) or approximate (identifiable) location of reported samples were incorporated in the database.

### Digital database GBIF

All georeferenced records for the genus *Boeckella* for the targeted study area were retrieved from the GBIF database on 30 July 2018. Records lacking precise geographic location (coordinates) were assigned georeferences by identification from the description of the reported collection locality included in the relevant metadata. The species list was updated to exclude erroneous or suspect records, rule out possible synonymies and include current taxonomy.

### Data Resources

The data underpinning the analysis reported in this paper are deposited at GBIF, the Global Biodiversity Information Facility (Maturana et al. 2018).

## Results

### Database Summary

A total of 815 unfiltered records were retrieved from all sources combined. Of these, 380 records were from the targeted study area (Maturana et al. 2018, https://doi.org/10.15468/zc6y59). Most records (278) were obtained from the published literature, followed by new sampling records (62), which represented more than 15% of the dataset analyzed. The GBIF database contributed further 40 records.

### Dubious records

*Boeckellasilvestrii* Daday, 1901, described in South America, has also been reported by GBIF in the South Orkney (https://www.gbif.org/occurrence/1056439704) and South Shetland Islands (https://www.gbif.org/occurrence/1056871457). Previously, Harding (1941) reported *B.silvestrii* in the South Shetland Islands, now attributed to *B.poppei* (Pugh et al. 2002). Therefore, the identification of *B.silvestrii* in the South Orkney Islands could be the result of a repeated source of confusion from this previous erroneous identification.

*Boeckellalongicauda* (Daday, 1901) has only been reported in the literature from southern Argentina (Menu-Marque et al. 2000), but there is a unique record from South Georgia, which is deposited in the Natural History Museum of London. This record is likely to be an erroneous identification and may correspond to *B.poppei*, as Bayly (1992a) commented that the morphology of the fifth leg of the male of *B.longicauda* is very similar to *B.poppei*, and probably the individual that Daday reviewed could be a variant of *B.poppei* (Bayly 1992a). Daday’s review of the genus retained this species mainly on the basis of the shape of the female’s urosome, which is very different from the morphology of *B.poppei*. It is also worth noting that *B.longicauda* was the only *Boeckella* species from southern South America that was not included in the phylogeny of Adamowicz et al. (2007). It is therefore important that the validity of this species be confirmed using both morphological and molecular techniques.

### Species richness

**Figure 1.**
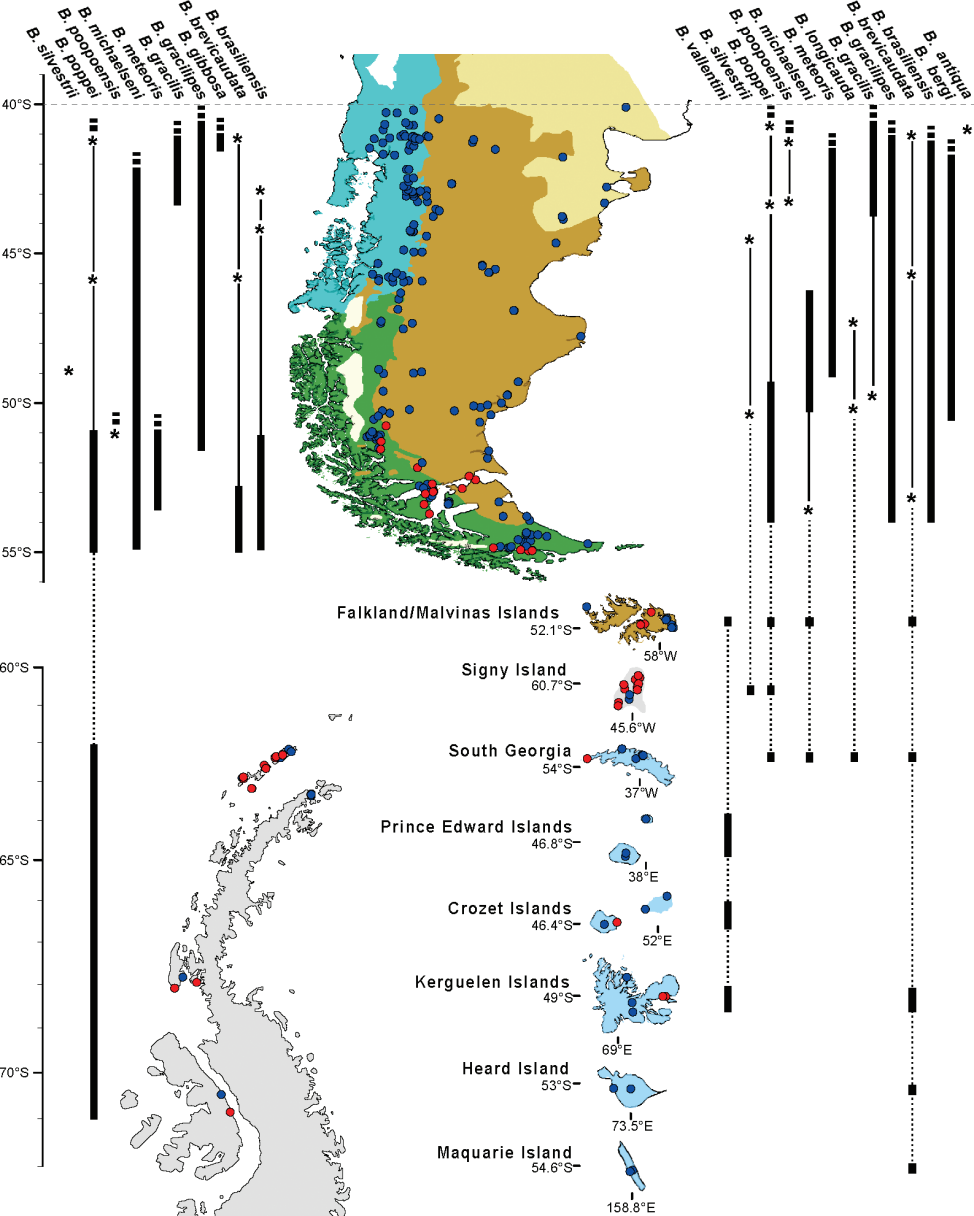
Spatial distribution of 14 *Boeckella* species from the targeted study area. The western (left side) and eastern (right side) of southern South America (green/blue: subpolar forest; brown: grassland), sub-Antarctic islands (light blue) and maritime Antarctic regions (light grey) obtained from records (red: obtained from field sampling; blue: obtained from literature and GBIF database) of all combined data sources. *: discrete outlier records; dash bars: distribution extended north of 40°S; dashed lines: geographic discontinuity. Records from East Antarctic were not included.

Fourteen species were recorded across the targeted study area (Fig. 1). Southern South America, including the Falkland/Malvinas Islands, contributed the highest number of records (297) and species richness (14) followed by the sub-Antarctic islands (34 and 5), South Orkney Islands (14 and 2), South Shetland Islands (23 and 2) and finally a single species (*B.poppei*) in continental Antarctica. Almost all records of *B.poppei* in Antarctica were from the maritime Antarctic (45), with seven records from the western Antarctic Peninsula (islands in Marguerite Bay and South Peninsula between 67°47'S; 68°54'W and 71°20'S; 68°17'W), and only two records from the continental Antarctic (Prince Charles Mountains, Enderby Sector). Although there was a small difference in species richness between the sub-polar forest (10 species) and grassland ecoregions (13 species) in southern South America, there are fewer records (102) from the latter region than from the forest (195).

Within the grassland ecoregion, four species were reported from the Falkland/Malvinas Islands (*B.brevicaudata* (Brady, 1875), *B.michaelseni* (Mrázek 1901), *B.poppei* and *B.vallentini* Scott, 1914), of which only *B.vallentini* is not shared with continental South America, rather being found on the sub-Antarctic Prince Edward Islands, Crozet Islands and Kerguelen Islands (and notably not South Georgia, the geographically closest sub-Antarctic island). All other species reported from the Falkland/Malvinas Islands are also reported from at least one of the sub-Antarctic islands (Kerguelen, Heard, South Georgia and Prince Edward Islands). South Georgia had the highest number of records (16) and species (4) of any sub-Antarctic island.

**Figure 2.**
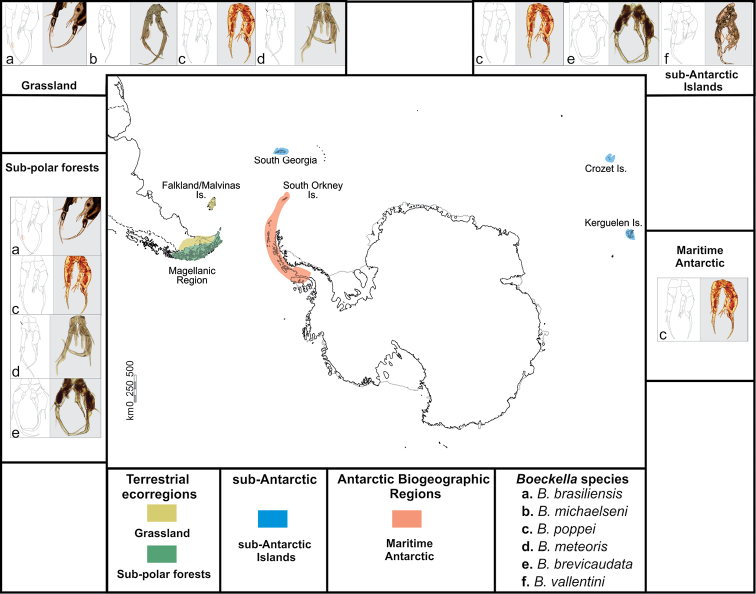
Map of the sampling locations in South America, sub- and maritime Antarctica. Six species were identified following the traditional taxonomic key (Bayly 1992a). *Boeckellapoppei* is present across the three Antarctic biogeographic regions. Drawings of the fifth male leg are modifications from Bayly (1992a).

Based on our sampling data, we identified six species distributed mainly in southern South America (*B.brevicaudata*, *B.meteoris* Kiefer, 1928, *B.poppei*, *B.brasiliensis* (Lubbock, 1855)), the Falkland/Malvinas Islands (*B.michaelseni*) and the sub-Antarctic islands (*B.vallentini*), adding 62 new records to the existing data (Fig. 2). These new records are generally consistent with the existing literature and GBIF data, with the exceptions of (i) new records reporting *B.brasiliensis* in Sierra Baguales and the surroundings of Punta Arenas, (ii) *B.brevicaudata* in Otway Sound, iii) *B.meteoris* in Tierra del Fuego, and (iv) *B.poppei* in Puerto Natales, Yendegaia National Park, Tierra del Fuego and Brunswick Peninsula in the Magallanes region, Robert and Greenwich Islands in the South Shetland Islands in northern maritime Antarctica, and finally the southernmost locality of Fossil Bluff on Alexander Island (71°20'S; 68°17'W). *Boeckellapoppei* is known to occur slightly further south in the same geological formation on southern Alexander Island in pools at Mars and Ares Oases (71°50'S; 68°15'W), which represent the true known southern limit of this species (P. Convey pers. obs.), but these records have not been formally published.

## Discussion

Increasing availability of data and the application of new molecular biological analyses and modeling techniques have generated the need for revision of the geographic distribution of many taxa. The present compilation and classification of *Boeckella* records represents a contribution to biodiversity knowledge and to the biogeographic distribution of members of the genus across three large-scale biogeographic regions in Antarctica and two ecoregions in southern South America. It is also appropriate to note here that recent research has recognized that the long-used three region classification of Antarctic terrestrial biogeographic regions does not expresses the full regional complexity of terrestrial biogeography in Antarctica, with 16 “Antarctic Conservation Biogeographic Regions” now recognized within the continent, five of which are contained in the maritime Antarctic as considered in the current study (Terauds et al. 2012; Terauds and Lee 2016).

Six of the 14 species of *Boeckella* occurring at high latitudes recorded in this study have been reported as exclusively distributed south of 40°S in South America (*B.brevicaudata*, *B.vallentini*, *B.silvestrii*, *B.antiqua* Menu-Marque & Balseiro 2000, *B.michaelseni* and *B.longicauda*Daday 1901). In particular, *B.silvestrii*, reported from 44° to 50°S on the Argentine Patagonian Plateau (Menu-Marque et al. 2000), and *B.antiqua*, which has never been recorded in any location since its original description from an ephemeral pond in Argentine grassland (Menu-Marque and Balseiro 2000; Pérez et al. 2012; Garcia and Dieguez 2014). In contrast, other species showed much wider distributions, in particular *B.poppei* which has been reported across the three Antarctic biogeographical zones (sub-Antarctic islands, maritime and continental Antarctic), as well as in southern South America.

The distribution of *B.poppei* is exceptional within the genus, including the Andean Region in South America (Morrone 2006) and the three biogeographic regions in Antarctica. Furthermore, the distribution of this taxon is unique in the Antarctic terrestrial and freshwater fauna. This species thus provides an important opportunity to evaluate possible historical and contemporary dispersal across major continental biogeographic provinces (see also Chown and Convey 2007). Pugh et al. (2002) suggested that the presence of *B.poppei* in Beaver Lake (eastern continental Antarctica) might be the result of an anthropogenic introduction, and more generally that all maritime and continental Antarctic non-marine crustaceans may have reached these regions through recent introduction events associated with human activities. However, several palaeolimnological studies of lake sediments have confirmed that this species has been present in both the maritime and continental Antarctic regions for up to 9000 years (Jones et al. 2000; Bayly et al. 2003; Bissett et al. 2005).

The two ecoregions examined in southern South America were the richest in terms of number of species and records available (14 and 297, respectively), followed by the sub-Antarctic islands (5 and 34), the maritime Antarctic (2 and 47) and finally the continental Antarctic (1 and 2). There is an important geographic gap in available records between the western Antarctic Peninsula and Enderby Sector in continental Antarctica. In a recent review of freshwater fauna in the south polar region, Dartnall (2017) reported only one record of *Boeckella* sp. in the region between Queen Maud Land (Schirmacher Oasis) and McMurdo Sound, including the Victoria Land Dry Valleys (Hansson et al. 2011). Although this seems to represent a low sampling effort, in reality, few, if any, suitable freshwater habitats are known to exist across this region today. For example, Hodgson et al. (2010) found the lowest species diversity yet observed in Antarctic lakes in the Dufek Massif and Shackleton Mountains, at the base and east of the Weddell Sea.

The presence of *B.vallentini* in the Falkland/Malvinas Islands and several sub-Antarctic islands (Kerguelen, Heard, South Georgia and Prince Edward Islands), but not in continental South America, must be noted (Table 1; Menu-Marque et al. 2000; Maturana et al. 2018). In the absence of molecular analyses, it is currently not possible to determine the phylogeographic relationship between these populations, and hence whether the Falkland/Malvinas acted as a source for current sub-Antarctic populations or vice versa. However, this is one of few known examples of the Falkland/Malvinas hosting terrestrial species that occur only from locations further south (i.e. sub-Antarctic and Antarctic regions). A second example is the terrestrial and supralittoral oribatid mite *Alaskozetesantarcticus* (Michael 1903), which occurs on sub-Antarctic South Georgia and throughout the maritime Antarctic, but not in South America (Block and Convey 1995).

**Table 1.** List of the 14 species of *Boeckella* considered in this study with their distribution in the targeted study area. *: Confirmed occurrence, **: Dubious record

Species	South America	Falkland/Malvinas Islands	Sub-Antarctic Islands	Antarctica
*Boeckellaantiqua* Menu-Marque & Balseiro, 2000	*			
*Boeckellabergi* Richard, 1897	*			
*Boeckellabrasiliensis* (Lubbock, 1855)	*			
*Boeckellabrevicaudata* (Brady, 1875)	*	*	*	
*Boeckellagracilipes* Daday, 1901	*			
*Boeckellagracilis* (Daday, 1902)	*			
*Boeckellagibbosa* (Brehm, 1935)	*			
*Boeckellalongicauda* Daday, 1901	*		**	
*Boeckellameteoris* Kiefer, 1928	*			
*Boeckellamichaelseni* (Mrázek, 1901)	*	*	*	
*Boeckellapoopoensis* Marsh, 1906	*			
*Boeckellapoppei* (Mrázek, 1901)	*	*	*	*
*Boeckellasilvestrii* Daday, 1901	*		**	**
*Boeckellavallentini* (T. Scott, 1914)		*	*	

The complexity of the morphology in this family of crustaceans, along with apparent plasticity in the diagnostic characters, can clearly lead to errors and considerable taxonomic and nomenclatural confusion (e.g. Bayly 1992a; Pugh et al. 2002). For example, Menu-Marque (2003) described *Karukinkafueguina* as a new genus and new species, but Adamowicz et al. (2007), in their study of Centropagidae phylogeny, found that *K.fueguina* genetically corresponds to *B.poppei* and concluded that *K.fueguina* is an aberrant version of *B.poppei*. Application of molecular systematics approaches to this group is required to limit misidentification, detect the existence of cryptic species, and assess the correspondence between currently recognized morphospecies and molecular evolutionary units. To date, two studies on Centropagidae integrating morphological and genetic data are available (Adamowicz et al. 2007; Scheihing et al. 2009). However, neither addressed material from the Falkland/Malvinas Islands, sub-Antarctic islands or Antarctica. In the near future, such molecular studies should allow evaluation of different biogeographical scenarios regarding the origin of the contemporary freshwater biota in Antarctica. In this context, this study provides the first revision and comprehensive description of a major part of the geographic distribution of the genus *Boeckella.*

## Supplementary materials

Supplementary material 1 Occurrences of *Boeckella* at high latitudes in the southern hemisphere. v1.1. Data type: ocurrence dataset This dataset is made available under the Open Database License (http://opendatacommons.org/licenses/odbl/1.0/). The Open Database License (ODbL) is a license agreement intended to allow users to freely share, modify, and use this Dataset while maintaining this same freedom for others, provided that the original source and author(s) are credited.Claudia S. Maturana, Sebastián Rosenfeld, Javier Naretto, Peter Convey, Elie Poulin
